# Does Organelle Shape Matter?: Exploring Patterns in Cell Shape and Structure with High-Throughput (HT) Imaging

**DOI:** 10.24918/cs.2022.3

**Published:** 2022-01-27

**Authors:** Carlos C. Goller, Graham T. Johnson, Kaitlyn Casimo

**Affiliations:** 1 Department of Biological Sciences, North Carolina State University; 2 Biotechnology Program, North Carolina State University; 3 Allen Institute

## Abstract

Organelle structure has been studied and visualized for decades; however, publicly available databases that use improved high-throughput microscopy of gene-edited cell lines have recently revolutionized the amount and quality of information now available for use in undergraduate classes. This lesson demonstrates how the use of high-throughput (HT) microscopy has generated data describing organelle structure and variability. Students access, analyze, and evaluate cell structure images using the Allen Institute for Cell Science’s Allen Cell Explorer. Students synthesize the information to make recommendations and propose a future experiment. Using web-based tools and a realistic scenario that merges antimicrobial drug screens with eukaryotic cell perturbations and structure, this case study provides a guided tour of the powerful applications of high-throughput microscopy.

## Graphical Abstract

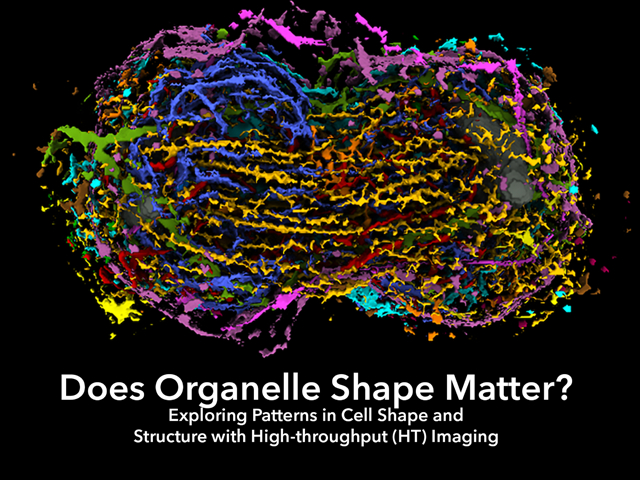

## INTRODUCTION

New genetic engineering and microscopy techniques allow researchers to visualize live cells with minimal disturbance and unprecedented clarity. High-throughput microscopy (HT) techniques have also evolved recently to incorporate improved robotic automation that can care for cells and generate microscope image data on par with images carefully collected by humans. These techniques have produced massive amounts of reproducible cell image data that have enabled novel types of cell biology analyses to be performed robustly. For example, these techniques have allowed for the creation of a benchmark for mean intracellular structure organization and the natural range of organizational variation from cell-to-cell (1) as well as for machine learning algorithms to predict changes in cell structure and function (2). This type of data has also proven useful in teaching virtual labs on mitosis (3). In our new lesson, students will learn about HT imaging and cell structure variation by exploring resources produced by the Allen Institute for Cell Science, which are freely available on the web.

The endoplasmic reticulum (ER) is a large organelle found inside of eukaryotic cells. It is a membrane-bound compartment with an extremely complicated organic morphology (anatomical shape) that varies greatly throughout different regions of the cell’s interior, across different stages of the cell cycle, and among cells exposed to different environmental conditions. The ER serves critical roles in cellular function, such as calcium storage, protein synthesis, and lipid metabolism (4,5). Therefore, despite decades of research, ER structure and associated diseases remain an active area of study (6).

The ER is introduced early in science education and revisited in undergraduate curricula, where the complex morphology is often depicted using only highly stylized illustrations or cross-sections that come from electron microscopy. Published educational resources most often engage students in thinking about cell organelles, along with their structure and functions, using debates and image libraries (7, 8). These approaches provide a useful foundation for understanding an organelle as complicated in morphology and function as the ER. However, the use of microscopy to gain insights into organelle structure-function relationships continues to be taught using idealized images or limited microscopy data that can create misconceptions (9). Using high-throughput microscopy images to show many examples of the ER can help students understand the relationships between diverse intracellular structures and functional information provided by the instructor. It provides an opportunity, relatively early in their learning, to develop an intuition for the diversity and variation of cellular structure, before students try to interpret more “real world” images in the lab or results from more sophisticated quantitative analysis techniques. This lesson guides students through the exploration of cell morphology to highlight the variability in cell populations and cycles. Using numerous microscopy images, students are able to gain a deeper understanding of what the ER truly looks like and how its structure varies.

Building on the *CourseSource* lesson created by Shelden et al. (3), this lesson uses the case study approach and structured group discussions to guide exploration of a challenging laboratory scenario and complex HT imaging procedures. The activity uses active learning and evidence-based practices. The lesson is part of a series of cases produced by the NSF High-throughput Discovery Science & Inquiry-based Case Studies for Today’s Students (HITS) group with the goal of using the case study approach to engage students in authentic inquiry of novel high-throughput datasets. “*Does Organelle Shape Matter?*” is designed for upper-division undergraduate students to explore a large image dataset, reflect on the impact of current and emerging HT approaches, and explore how HT approaches are contributing to our fundamental understanding of the cell.

### Intended Audience

This lesson was designed for juniors, seniors, and early graduate students from diverse majors and programs at a large public research university. We have taught this lesson to students in chemical engineering, biological sciences, biomanufacturing, microbial biotechnology, and physiology concentrations. This lesson could also be useful early in their academic career for students in programs for microbiology, molecular/cell biology, or pre-medicine/pre-health majors.

### Required Learning Time

In-class time required for this lesson is approximately 1–2 hours. As a potential alternative, an entirely online asynchronous version of this lesson would require time to complete the case study worksheets and follow instructions, totaling approximately 2–3 hours. A 15–30-minute subsequent wrap-up and discussion session led by the instructor is encouraged to emphasize key points, address any misconceptions, and share findings.

### Prerequisite Student Knowledge

Successful implementation of this lesson is not dependent upon particular background knowledge and can be adapted to many contexts by the instructor. Knowledge of basic cell biology, genetics, and microbiology is recommended. Specifically, it is recommended that students know about ER function, fluorescence microscopy, and CRISPR gene editing. A brief overview of the Allen Cell Explorer is available as a video: Allen Cell Explorer Tutorial: Navigating the 3D Cell Viewer (3:31 min.). The entire activity is web-based. Students are expected to already understand basic concepts of fluorescence microscopy and gene editing, though no detailed methodological knowledge is required. In this lesson, they will focus primarily on learning about the endoplasmic reticulum and the general concept of natural cell structure variation using HT microscopy images.

### Prerequisite Teacher Knowledge

Teachers should have a working knowledge of cell biology, molecular biology, genetics, and be comfortable using the Allen Cell Explorer website. By completing our lesson prior to instruction, teachers will be able to test their level of comfort with the site and its tools. Teachers should also have familiarity with the spinning disk confocal fluorescence microscopy techniques used by the Allen Institute for Cell Science. Teachers are encouraged to view the video tutorials provided on the site and the Haupt video tutorial on endogenous fluorescent tagging of cells using CRISPR (10). While a key is provided, several questions have alternate reasonable responses, and instructors are encouraged to solicit different student responses for discussion.

## SCIENTIFIC TEACHING THEMES

### Active Learning

This lesson uses a case study that can be completed by students individually or in groups, with class-level wrap-up discussions and individual metacognition reflections. The case study pedagogy is considered a student-centered active learning pedagogy by the President’s Council of Advisors on Science and Technology (PCAST) report (11) and shown to improve student performance even when cases were authored by others (12). Group worksheets have been shown to have a positive impact on learning gains of undergraduates (13). Furthermore, case studies can be used as homework assignments (completely asynchronous and online, for example, 14), containing realistic scenarios that invite students to explore data and arrive at conclusions before larger group discussions (15). Questions used in this case study can be adapted to be answered using student response systems (“clicker questions”) or are suitable for think-pair-share active learning techniques even in distance learning situations. For suggestions and possible modifications, please see Supporting File S3. Does Organelle Shape Matter? - Teaching Notes.

### Assessment

Students submit answers to the case studies in small groups and reflect on the activity individually. Case study submissions can be reviewed by instructors and graded for accuracy, effort, and completion. We encourage instructors to review the case study as a group debrief, inviting participation and contributions by all members. Students self-evaluate their learning in their reflections. Individual student written reflections encourage students to think about the lesson and connect it to current knowledge and future applications, practicing metacognition.

We encourage instructors to provide suggested responses (Supporting File S2. Does Organelle Shape Matter? - Teacher Key) as a posted document. The use of de-identified student quotes and example work has been approved by North Carolina State University IRB under protocol number 117558.

### Inclusive Teaching

This lesson was designed to be approachable to and accessible by a broad range of learners in order to expose students to high-throughput datasets and their potential. Case studies can be adapted to a variety of courses and formats. We have implemented this case as a series of handouts formatted for ADA accessibility to be screen-reader friendly. We provide the worksheets both as physical handouts and as editable electronic documents. We have used Google Docs to encourage groups of students to work collaboratively, both in class and virtually (often asynchronously). In-class discussions are used to facilitate contributions by all participants. Several of the questions have multiple potential responses and can be used to foster productive discussions about the methodology and future experiments, prompting students to think critically about high-throughput technologies, for example. See Supporting File S3. Does Organelle Shape Matter? - Teaching Notes.

Additionally, in-class administration of the case can be accomplished using several student-centered active learning techniques including group work, think-pair-share, and whip around to promote learning (15). Case studies can be used as low-stakes assignments to foster productive discussions, use of high-throughput databases and resources, and quantitative skills. Instructors are encouraged to allow students to submit completed cases in alternative formats including audio and video files.

## LESSON PLAN

This lesson is designed for a 75-minute class period (see Table 1 for the progression through the lesson with approximate timing). The intention of this lesson is to address common misconceptions about cell organelle structure and raise awareness of the methods and applications of high-throughput (HT) technologies. Students may think of cell morphology and organelle shape as 2D static structures when these are dynamic and three-dimensional in shape. By exposing students to thousands of authentic microscopy images and high-throughput approaches, students learn how our knowledge of cell structure has evolved. This case exposes students to large microscopy datasets available on the Allen Cell Explorer website. The lesson uses a case study highlighting the tools used to create these datasets and the numerous applications of HT technologies. Using this lesson, students interact with datasets produced using HT approaches to answer fundamental questions about cell biology and drug discovery.

### Pre-Class Preparation

We encourage the instructor to become familiar with the Allen Cell Explorer web site and read the case study and instructor key before implementing the case. First, instructors should assign students to small groups (3–4 participants per group). Instructors should also emphasize that the teams will need to work collaboratively to share their findings and reach conclusions that will later be shared in a class-level discussion. The goal is to foster an environment that encourages active participation and discussion by all participants.

We begin the lesson by explaining the goals of the case study, its format, and the datasets that the students will have the opportunity to explore (S3. Does Organelle Shape Matter? - Teaching Notes.). It is important to highlight how the case study will introduce high-throughput microscopy and the use of open datasets and public resources. We ask students to think about what high-throughput microscopy means to them, and how it could be applied.

The instructor may consider stopping after each part of the case study to request student responses and encourage class discussion. Random calling after providing sufficient time for students to generate a response and the *whip-around* method for eliciting student contributions in an equitable manner are encouraged (13). However, we encourage instructors to be mindful of equity and the assumptions of sharing, as some students may not feel comfortable sharing in large groups due to anxiety, stereotype threat, and other factors (16).

### Progressing Through the Activity

The students receive the case study provided in Supporting File S1. Does Organelle Shape Matter? - Student Handout. To help students progress through the activity, the instructor should check in with groups and provide assistance when necessary. Students may encounter difficulties finding and analyzing images from the Allen Cell Explorer. Instructors should provide assistance to groups and announce common sticking points to the class. An overview of the activities and example timeline are provided in Table 1. In Part I, students explore the typical structure of the endoplasmic reticulum (ER). There will be a variety of drawings and sketches, often based on the highly stylized ER structure provided in textbooks and Wikipedia, for example. In question 2, students will explain the function of the ER in their own words, asking them to do online research or activate prior knowledge from high school biology or college cell biology courses.

In Part II, students visit the Visual Guide to Human Cells. Students should read the information on the web page to learn how researchers visualized the ER. A helpful video tutorial of the Allen Cell Explorer is available (Allen Cell Explorer Tutorial: Navigating the 3D Cell Viewer). This part of the case familiarizes students with the protein tagging system used to visualize cell structures. Sec61-beta tagging is used to visualize the ER, for example. Students then use the guide to compare the ER in different cell cycle phases, learning that the ER structure often does not look like the images they may have encountered in textbooks or diagrams of cells.

Part III of the case uses web-based tools available on the Cell Feature Explorer to measure cellular and nuclear volumes of student-selected images of cells. Instructors should encourage participants to compare the values they obtained to start discussions about cell variability. The Integrated Mitotic Stem Cell is used to teach students about variability of other cell structures over space and time. The questions are designed to encourage curiosity and highlight what students find intriguing about the images (question 4). The methods used by the researchers at the Allen Institute are important to emphasize. Thus, question 5 asks students to navigate to the Methods for Microscopy page to learn more about the process used and summarize their interpretation in no more than five sentences. After completing questions 6 and 7, it may be useful to have groups of students compare responses. Instructors should ask groups to share what they can infer from the cells in the Allen Cell Explorer that we can’t do with the stained CHO cells. Then, instructors can ask about potential limitations of the endogenous fluorescence method used by the Allen Institute, such as alternative localization or compromised function. The desired outcomes of these discussions will be to compare approaches and have students describe the methodology and potential limitations. Instructors can then identify misconceptions and make sure all groups have an understanding of the methods used to generate this dataset.

Part IV of the case study returns to the implications of these approaches and primes students to develop a plan for next-step experiments. This set of questions encourages participants to think about high-throughput microscopy methods and their applications. The final question asks students to design a future experiment to follow up on the experiments in the case study.

The last section of the case is a reflection to wrap up the activity and prompt students to individually state the most memorable concepts or skills they learned with this activity and what they are left wondering about. The objective is for students to reflect on what they have learned and share with the instructor what they are still unsure about or are interested in learning more details about.

### Synthesis & Reflection

We encourage instructors to revisit themes from responses in a class-level discussion that highlights the methods used by the Allen Institute, applications and implications of high-throughput approaches, and the interconnectedness of cell biology and searches for antimicrobials using high-throughput screening. We announce to the class that we will revisit the case study during our next session together and to bring their completed case studies. We start the next class period with dedicated time (15–30 minutes) to have this discussion. We restate the learning objectives of the case study and then ask representatives from each group to summarize a part of the case to the entire class. We ask other groups if they had similar thoughts and why or why not. The goal is to review the main findings from the case study. At the end, we project the Visual Guide to Human Cells webpage and prompt students to share what they thought was the most memorable outcome of the case study. We tend to emphasize the size of the dataset and the opportunities to use these cell lines, images, and methods for other studies and comparisons. The take-home points we want students to leave with are: that the ER shape and size does vary, that this open dataset produced by high-throughput microscopy approaches highlights cell organelle variability and provides data for modeling tools, and that they brainstorm a future experiment based on what they learned.

Instructors should ask several groups to share their proposed experiments to illustrate different ideas. We also ask students to think about what they would do with the hiPSC cell lines if they were to run their own experiments. To engage in productive discussions, we advise novice instructors to state to the class participants that you will call on each group to share their thoughts on each part of the case study. Groups will have time to find their case studies, briefly share their thoughts, and assign a speaker. Groups are allowed to pass once but you would be expecting all groups to contribute. For the final discussion about future experiments, questions, and applications, all participants are encouraged to contribute their thoughts, by raising their hands, responding to poll questions, or other response systems. Instructors can address pinch points, or areas of difficulty for students, such as the use of tools on the Allen Cell Explorer site or the significance of the cell lines used for the study. Each group submits one completed case study document. The instructor provides feedback on this document. Students individually reflect on the case study by answering a reflection prompt.

## TEACHING DISCUSSION

There are a number of modifications that can be made to the case study to extend it and dig deeper into the methodology used, the significance of high-throughput approaches, variability in the structure of the endoplasmic reticulum (ER), and future experiments that can be performed to improve the therapeutic potential of the drug Dr. G’s group found.

Introductory students can engage in the analysis of authentic high-throughput datasets using the Allen Cell Explorer. The case can be modified to emphasize the variability of cell organelle structure and how high-throughput approaches allow researchers to obtain large datasets to build more accurate models. In courses with learning objectives focusing on the process of science and cell organelle structure, after implementation of the case study, instructors may use class discussions or online forums to search for published studies noting variability in endoplasmic reticulum staining or size under specific conditions, for example. Depending on the focus of the course, the lesson may also be extended to have students conduct the same analysis on a different cell structure.

A natural next step for advanced, computationally-oriented students is to explore the label-free method developed by the Allen Institute to process its HT data. In our experience, several upper-division students and graduate students were intrigued by the use of HT microscopy data from these engineered cell lines to create a label-free prediction system. Students could be assigned the video resource by Haupt et al. 2018 (10) or the Ounkomol et al. publication (17) describing the label-free prediction system for discussion in a subsequent class session. These two publications describe cutting-edge techniques that include CRISPR genome editing and high-throughput image analyses that may align with a number of cell biology and genomics course objectives. However, these publications are not open access and will require access via an institutional subscription.

We encourage instructors to emphasize the application of these high-throughput technologies to solve real-world problems, such as identifying novel antibiotics or understanding the impact of drug perturbations on cell morphology and function. The Allen Cell Explorer also has a small amount of data on cell images after drug perturbation.

In addition, in Spring 2020 we implemented the case remotely using Google Docs shared with each group. We were able to monitor the progression of the students on the case by viewing the document and adding comments when necessary. We were able to summarize the main highlights and learning objectives of the case using a short screencast that was shared with students. In the fall of 2020, we implemented in an asynchronous online course by posting the case to the course website. Students completed the case individually, and the class used forums for discussions. In both instances, the data analysis of the case is unaffected, as it relies exclusively on online data resources.

Representative student quotes are presented below highlighting what participants learned by completing this activity:

“The potential of a HT pipeline for image analysis and how it can expedite the research process… How useful the selected tags for each organelle are. Are they as selective as these images imply or is there some non-selectivity that has to be processed out? The best way to start this process would likely be to look through their website. Failing that, there are databases available that have compiled protein info and will show if these proteins have been found localized to other locations within the cell. ”

(Student, Fall 2020)

“Whereas the things that can be created with high-throughput technologies can be impressive and of equal quality to results from more arduous experiments, it does not mean that these approaches can answer each mildly-related question completely. The Allen Institute has a great number of high-resolution photographs that allow people to see different cell structures, giving us a better idea of cell morphology in general. However, a targeted question assessing a specific subcellular component may require the utilization of other technologies to yield relevant results, or at the very least add a layer of refinement to data already presented. It was clear in the case study that the ER, for instance, changes in volume. In an experiment, this makes it hard to predict whether changes are due to cell size or phase, or to a specific growth condition or drug addition. It would be difficult to address this completely by image analysis alone, even with machine learning. Even though impressive resources can be developed with HT protocols, it is important to realize that more work may need to be done in order to get impactful results for more specific questions.”

(Graduate Student, Fall 2020)

“High throughput technologies allow data to be generated at a much faster rate than could be done just by human researchers. A good example would be the imaging in the Case study. It would have taken much longer, or been much more expensive to make all those images without the use of HTP.… However, with the use of those technologies, the images were able to be completed in a very timely manner, which then allowed human researchers to analyze them and find trends.”

(Undergraduate Student, Fall 2020)

“Combination of using a sample of images that are more accurate but time consuming with methods that can be reproduced more quickly to produce prediction models isn’t something most of us had considered before. It is an interesting idea to get some accuracy but also prepare for experiments that could be done in a high throughput manner.”

(Group of Students, Spring 2020)

“I thought it was interesting to see how the textbook images aren’t always what they seem to be…or even accurate for that matter. It was also helpful to learn that there are other resources that can be used that can provide more reliable pictures/images…. Using the Allen institute microscope, I think it would be interesting to explore other organelles, and their functions.”

(Group of Students, Spring 2020)

“From this case study, it was important that we were able to learn about the approaches of high-throughput technology and the different applications that it has especially in exploring cell shape and structure. This methodology is extremely unique and being able to use this website is extremely useful and can have applications to our careers and future endeavors.”

(Group of Students, Spring 2020)

Based on student responses both individually and in groups, this case study prompted students to think about the applications of high-throughput microscopy and elicited interest and numerous additional questions. Responses by both undergraduate and graduate students highlight similar topics, as well as thoughts submitted by groups.

Possible modifications to an online implementation of this case include individual online written or verbal (using the video/audio tool of the LMS) reflections and posting to a class forum to share thoughts and future applications of the label-free prediction system created by the Allen Institute. Instead of assigning the case study as a worksheet, instructors could teach the lesson interactively in a class session, using clicker questions to reinforce concepts and assess achievement of the learning objectives in real time. It is important to provide students with the opportunity to practice skills and retrieve knowledge gained by completing this case study. Instructors can design short quizzes that address learning objectives focusing on the nature and applications of high-throughput technologies. Short essay questions can challenge students to research cell organelle variability or the use of cytotoxicity assay methods for drug screening and present evidence supporting a proposed experiment. Another variation could be to ask students to use the Allen Cell Explorer website to measure a reasonable number of cell organelles (~35) and present that data for subsequent quantitative analyses. This assignment would then allow students to practice the use of the Cell Explorer and obtain data for statistical analyses to support or reject their hypothesis that ER structure does vary (under × condition, for example). While the addition of quizzes and essays should be as low-stakes assessments, summative assessments can include questions identifying key concepts of the case on a final exam or unit evaluation.

## Supplementary Material

S1 S1. Does Organelle Shape Matter? - Student Handout

S2 S2. Does Organelle Shape Matter? - Teacher Key

S3 S3. Does Organelle Shape Matter? - Teaching Notes

## Figures and Tables

**Table 1.** Description of timeline and activities for the implementation of this case study.

Activity	Description	Estimated Time	Notes
**Preparation for Class**			
Prepare the case study to distribute in class	1. Make one copy of the Case Study Handout (Supporting File S1) for each group of 3–4 students or distribute it individually to students, if desired. 2. If an electronic version is used, copy and post the document for all students to access via the Learning Management System (LMS) and/or Google Docs. 3. For in-person classes, ensure each student group has a computer with Internet access to use the Allen Institute Cell Explorer website.	15–20 minutes to prepare enough sets for all groups in the class.	The Case Study is provided in Supporting File S1. A suggested script to introduce the lesson is included in S3. Teaching Notes - Suggested scripts, resources, and considerations for implementing and adapting this case. Highlight the objectives of the lesson and the use of web-based resources to explore large and novel high-content microscopy datasets. It is also recommended that the instructor become familiar with the Allen Cell Explorer website before the lesson. The Allen Institute has webinars available on the use of the site (Instructional Videos & Tutorials for Cell Methods, see Allen Cell Explorer Tutorials near the bottom).
**Class Session**			
Introduction	Introduce the learning objectives and context for the high-throughput microscopy case study.	10 minutes	Ask the class: What is high-content microscopy? What can high-throughput (HT) approaches enable us to do? Explain the expectations for completion of the case study worksheets, and encourage participants to use their devices to search for relevant reliable information when necessary.
Case Study	Students work individually or in groups to complete each part of the case study. The case study is divided into four segments, plus a reflection (below).	~45–60 minutes	Some may require assistance during parts of the activity. The instructor can assist individual groups, for example. In order to foster meaningful discussions at the class level, the instructor should pause between parts of the activity to review responses, soliciting contributions from participants. The instructor should provide sufficient time for all to finish each part of the case study.
Reflection	Students individually answer a reflection prompt before a class-level discussion.	15 minutes	The reflection prompt emphasizes the significance of **high-throughput approaches** and their applications with the goal of having students connect this knowledge to previous information and future applications. It is important for students to reflect on their experience after working through the case study.
Review	The class reviews their findings and discusses the relevance of high-throughput approaches to answering the questions posed in this case study.	15–30 minutes	The instructor should review all parts of the case at this point, highlighting additional resources when necessary.

## Learning Goals

### Students will:

- understand how cell organelle structure varies between and within cells, as well as over time, focusing on the endoplasmic reticulum (ER).
- learn about the real-world interpretation and application of high-throughput microscopy datasets and methodologies.
- learn how to apply open data as a reference to provide context for their own data.

## Learning Objectives

### Students will be able to:

- describe the morphologies (anatomical structure) of the endoplasmic reticulum (ER) and its primary functions.
- describe the purpose and applications of high-throughput microscopy.
- compare cell structures using general visual analysis tools, in this case, demonstrated by the 3D Cell Viewer on allencell.org.
- design a future experiment using similar data that builds on their findings.
