# Supplementary material for: Does Organelle Shape Matter?: Exploring Patterns in Cell Shape and Structure with High-Throughput (HT) Imaging
Source: CourseSource. Author manuscript; Available in PMC 2022 Aug 17. (PMC9385133; doi:10.24918/cs.2022.3)
Supplement: S1 — Does Organelle Shape Matter? - Student Handout [file NIHMS1777492-supplement-S1.docx]

**
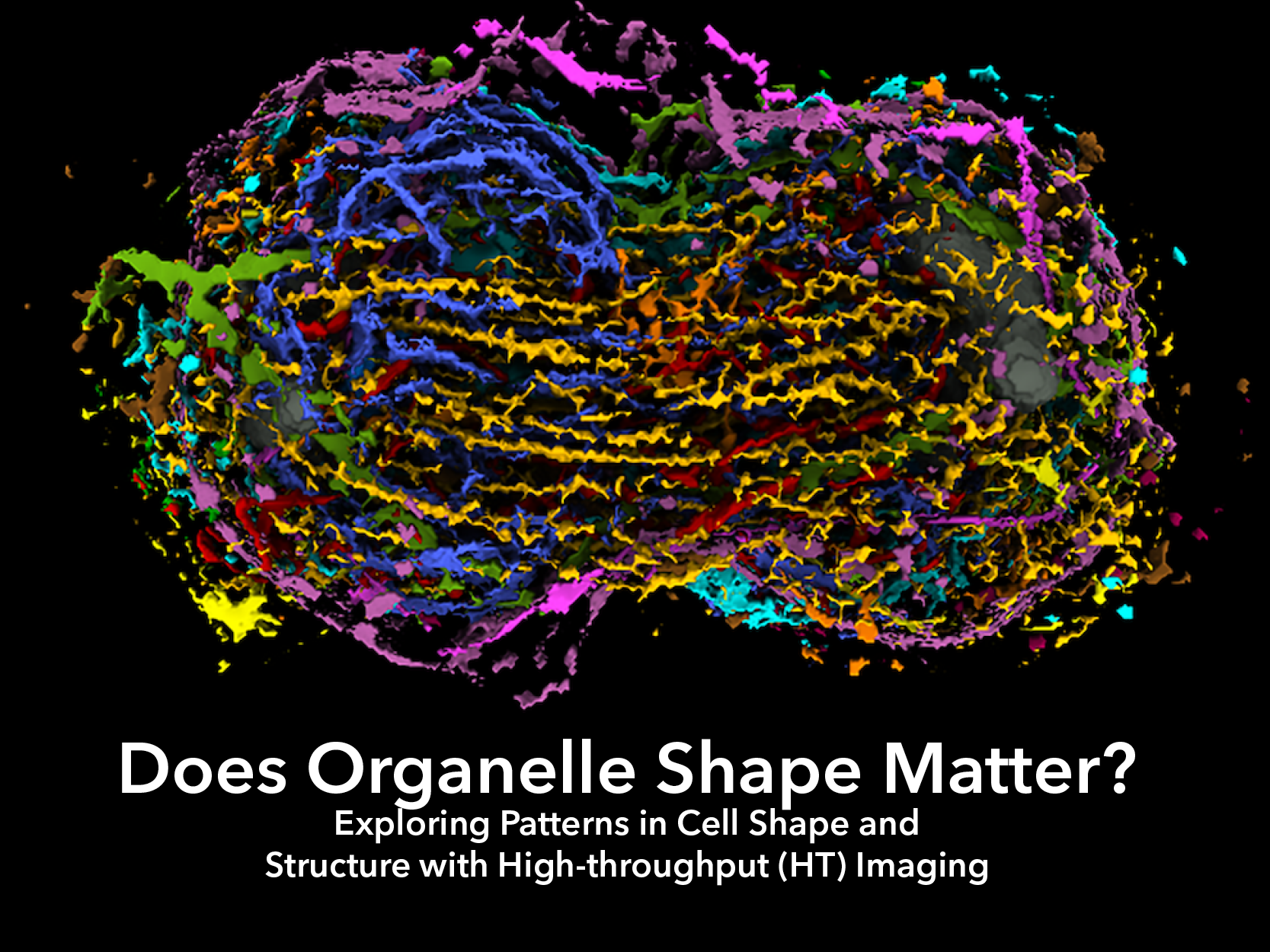
**

**Does Organelle Shape Matter:**

**Exploring Patterns in Cell Shape and Structure with High-throughput (HT) Imaging**

**Student Handout**

Carlos C. Goller (North Carolina State University), Graham Johnson (Allen Institute for Cell Science), and Kaitlyn Casimo (Allen Institute)

#

# **Learning Objectives**

1. **Define** the role of the endoplasmic reticulum
2. **Explain** the purpose and applications of high-throughput microscopy
3. **Compare** cell structures using the Allen 3D Cell Viewer
4. **Design** a future experiment to build on your findings

# **Part I. What has happened to these cells?**

Dr. G and his summer students want to discover a drug that could be used as an antibiotic against *Delftia acidovorans*, an opportunistic bacterial pathogen that has been found in drains, faucets, and water filtration systems. They used high-throughput (HT) drug screening to test a collection (“library”) of cancer drugs from the National Cancer Institute (NCI) and were excited to find a promising **hit**, a compound with potentially useful drug properties. This compound is highly potent at low doses against *Delftia acidovorans* and not cytotoxic when tested on mammalian cells. However, one of Dr. G’s students was learning from a friend in another lab how to use a stain to label the endoplasmic reticulum (ER) in live Chinese Hamster Ovary (CHO) cells, and she noticed that cells treated with low doses of their promising compound have **wildly** variable ER sizes and morphologies. She tells Dr. G., and together they search for information about ER structure. Dr. G. is a microbiologist and, along with his students, has limited cell biology experience.

Dr. G. and his students decide to use the Allen Cell Explorer open cell biology data as a reference dataset to compare to their drug-treated cells. This is a large, high-throughput dataset containing tens of thousands of cells. They hope to gain some useful insights about the human ER from this dataset.

## **Questions**

1. Draw a eukaryotic cell and identify the endoplasmic reticulum (ER). How is this structure different from a prokaryotic cell?
2. Demonstrate your knowledge: explain to the average adult the function of the ER in 1-3 sentences.
3. Demonstrate your knowledge: describe the shape of the ER you just drew.

#

# **Part II. This image does *not* look like the textbook image!**

Dr. G. and his students visit the Allen Cell Explorer [Visual Guide to Human Cells.](https://www.allencell.org/visual-guide-to-human-cells.html) This resource, along with other open data we will use in this case, has been produced by the [Allen Institute for Cell Science](http://cellscience.alleninstitute.org). They read about the Endoplasmic Reticulum (ER) and how researchers visualized the ER by **labeling Sec61-beta proteins** as discussed below.


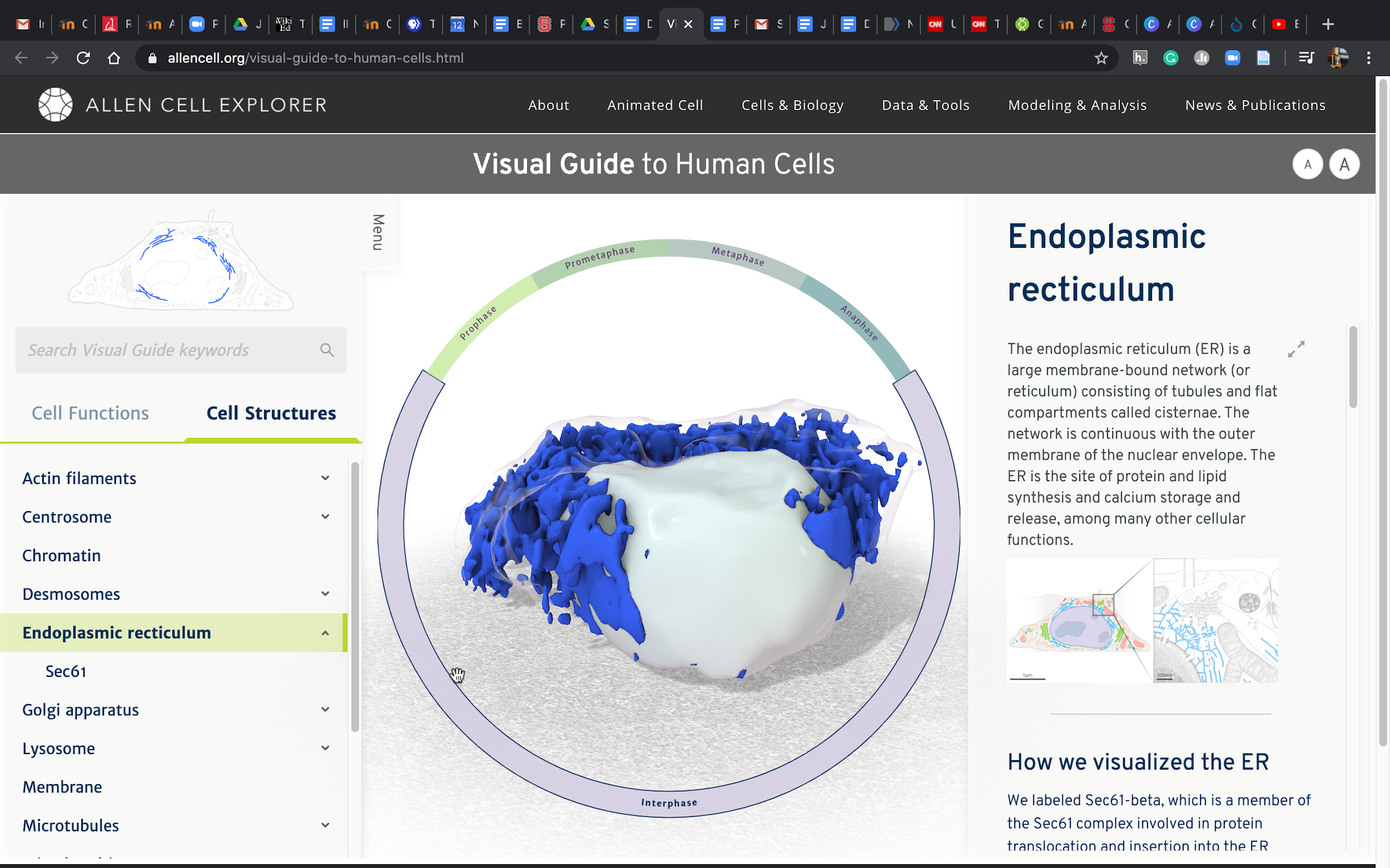


They then decide to view cells using the [**Allen Cell Feature Explorer**](https://cfe.allencell.org/) and visualize cells with the **Sec61-beta** protein tagged as shown next. Watch this 3-min [video](https://www.youtube.com/watch?v=UKXtEddLzjg) to learn more about the 3D Cell Viewer. These features are found in the Cell Feature Explorer, along with some additional tools for quantitative analysis of the cells. We’ll be concentrating on the cell images, but you may find additional insights using the graphing panel.

##

## **Your turn!**

Help Dr. G. and his students by visiting the Allen Cell Explorer resources. With the information you find, answer the questions below.

## **Questions**

1. Using the [Visual Guide to Human Cells](https://www.allencell.org/visual-guide-to-human-cells.html), look at the shape of the ER during the different phases of the cell cycle (prophase, prometaphase, metaphase, anaphase, and telophase/cytokinesis). Click on the **Menu** tab to reveal the cell structures and select ER. You can click on the **name** of the phase, and the visuals will change. You can drag the cell visual to reveal different perspectives. What changes do you notice? (in 2-3 sentences)
2. You notice under the **Morphology** section (panel on the right) the following text: “In hiPS cells, the ER is localized to the nuclear periphery and in tubules and sheet-like structures throughout the cytoplasm”. Dr. G’s students saw fluorescence using the ER-specific tag that **varied** in shape both within and between different cells. What explanation do you have for this? (2-3 sentences)
3. You then navigate to the [Allen Cell Feature Explorer](https://cfe.allencell.org), select **Sec61-beta** under Protein Tag from the menu on the left, and select several dots from the plot corresponding to cells. Images of cells will appear in your Gallery on the right, and you can click to visualize cells with fluorescently tagged Sec61-beta.

You click on a couple of cell images, keeping in mind that the green signal corresponds to a fluorescent tag for the ER, and the membrane and DNA are also stained. Do you notice any patterns? (2-3 sentences).

# **Part III. Different looking cells? Let’s get quantitative.**

You have been looking at cells for some time now and notice a wide **variety of patterns** for the Sec61-beta tagged cells. Furthermore, **it seems all cells look different!** This wasn’t what you are used to from your Cell Biology class in college... You want to quantify this as best you can.

In the Cell Feature Explorer, scroll back up to the plot to start measuring the cellular and nuclear volumes of the ER (tagged using the Sec61 protein tag).

## **Tasks & Questions**

1. Measuring cellular and nuclear volumes. Select **three cells** and compare the ER volumes. Record the approximate values.

|  | **Cellular Volume** | **Nuclear Volume** |
| --- | --- | --- |
| **Cell 1** |  |  |
| **Cell 2** |  |  |
| **Cell 3** |  |  |

1. Compare your measurements with those obtained by other group members.

You want to learn more! You find: [The Integrated Mitotic Stem Cell](https://imsc.allencell.org/).

… and carefully scroll down and read… You use the 3D Cell Viewer embedded in the Integrated Mitotic Stem Cell page to visualize the ER and other structures superimposed in space and time.

##

## **Questions**

1. Do these images help you explain the variability in ER volumes you recorded previously? Think about the cell cycle and variability of other organelles.
2. What do you find intriguing about the [The Integrated Mitotic Stem Cell](https://imsc.allencell.org/) web page and data?

You start to wonder... **How are they visualizing the ER and other organelles?** You navigate to the [Methods for Microscopy page](https://www.allencell.org/methods-for-microscopy.html) to learn more about the process used by the Allen Institute and begin to read...

##

## **Questions**

1. Read the [Methods for Microscopy page](https://www.allencell.org/methods-for-microscopy.html). Summarize the methods used in no more than **five** sentences.


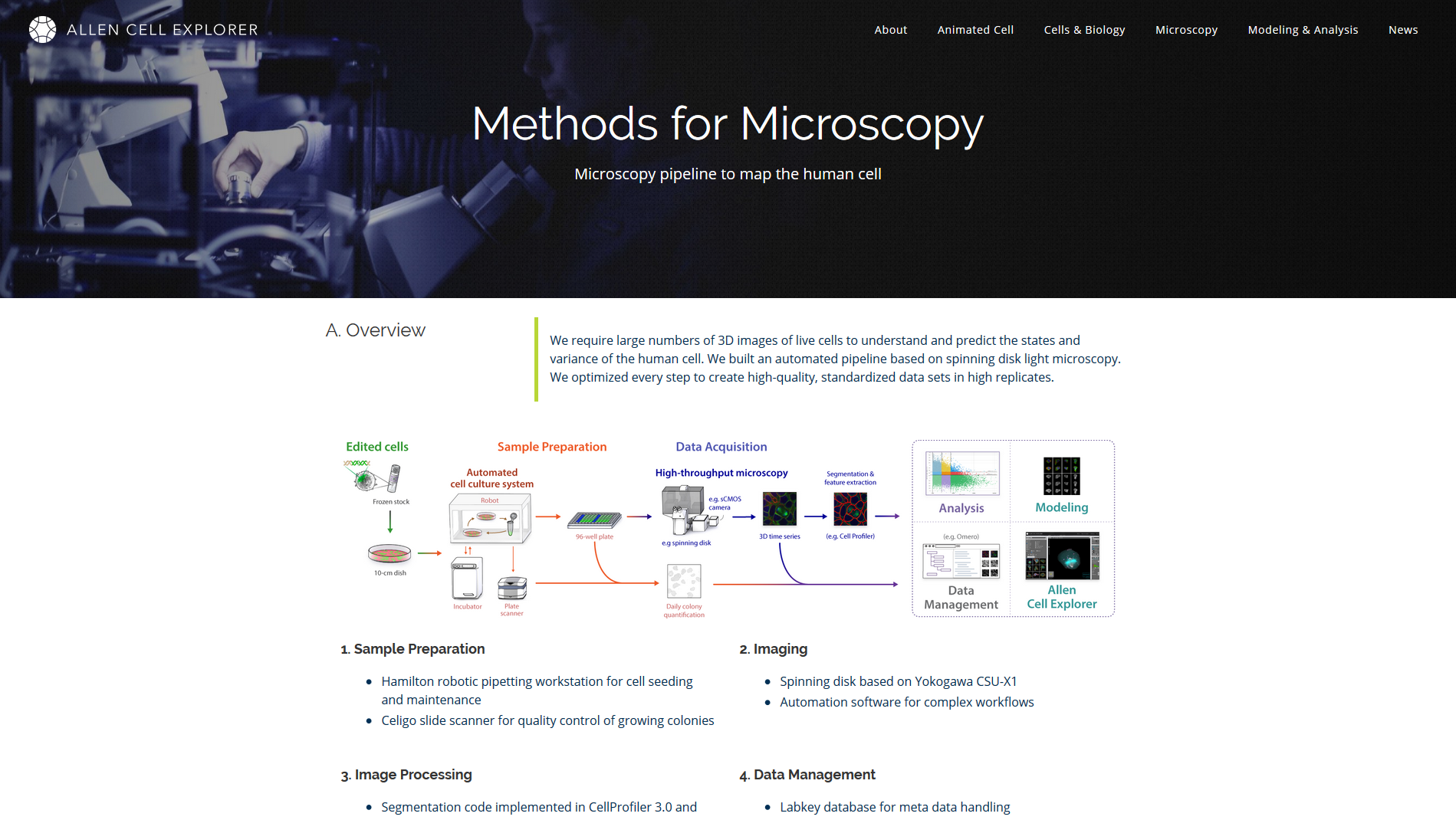


1. Think about Dr. G’s drug-treated CHO cells. What can we infer or visualize from the cells in the Allen Cell Explorer that we can’t do with the stained CHO cells?
2. What is one limitation of the endogenous fluorescence method used by the Allen Institute?

#

# **Part IV. Does this make sense, Dr. G?**

You have learned *a lot* from helping Dr. G’s students and visiting the Allen Cell Explorer. However, you still have to report back to Dr. G!

## **Questions**

1. Think about the **high-throughput microscopy** approach used by the Allen Institute and the tools developed to measure cell structures. What did you learn about the ER from the tools and site?
2. Think about the cells you viewed, what you learned about the ER, and the *Delftia* experiment. Do you think this high-content imaging approach will help Dr. G’s students? Why or why not? Think about the knowledge gained from this **approach** and the limitations of the dataset.

1. The reference data from the Allen Cell Explorer comes from human induced pluripotent stem cells, but Dr. G. and his student were using CHO cells in their drug screening study. What similarities are there between these two types of cells? What differences? Would you expect systematic variation between these two types of cells? Would you expect CHO cells to be more similar to hiPSCs than yeast cells are? Why?
2. If you could work with researchers at the Allen Institute, what would you do next? Design an **experiment** to build on your findings and help Dr. G. Describe the goal of the experiment, the methods and resources you will use, and the expected findings. Think critically about the potential limitations of your approach. Explain the experiment you design in 5-10 sentences. You may need to use external resources such as PubMed to find background research related to your experimental design.

#

# **Reflection**

1. What was the most **memorable** concept or skill you learned from this case study?
2. What are you left **wondering** about [“**Nothing**” is an unacceptable answer]? How do you start the process of learning the answer?
