# Supplementary material for: Does Organelle Shape Matter?: Exploring Patterns in Cell Shape and Structure with High-Throughput (HT) Imaging
Source: CourseSource. Author manuscript; Available in PMC 2022 Aug 17. (PMC9385133; doi:10.24918/cs.2022.3)
Supplement: S2 — Does Organelle Shape Matter? - Teacher Key [file NIHMS1777492-supplement-S2.docx]

**
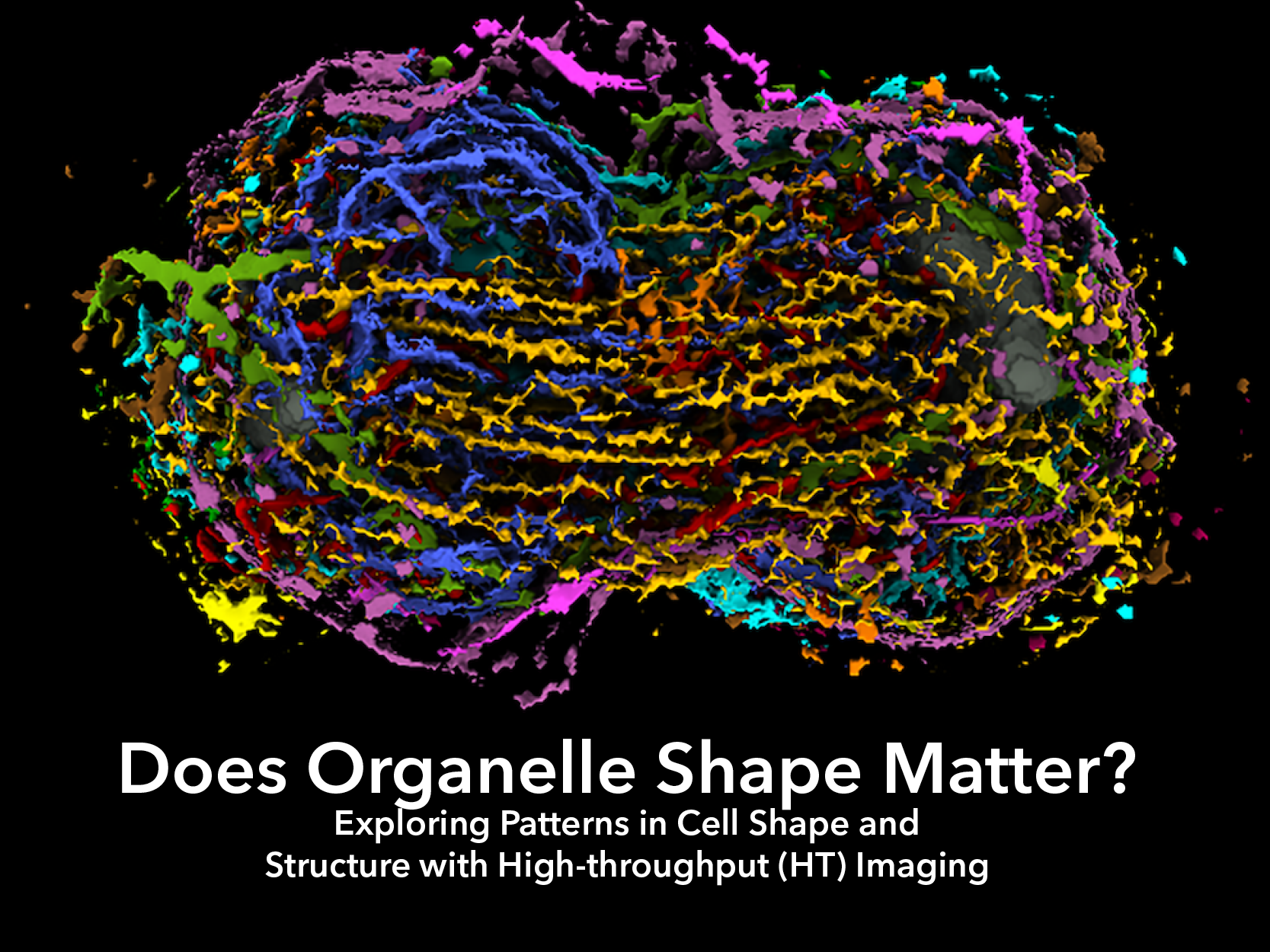
**

**Does Organelle Shape Matter:**

**Exploring Patterns in Cell Shape and Structure with High-throughput (HT) Imaging**

## **Teacher Key**

Carlos C. Goller (North Carolina State University), Graham Johnson (Allen Institute for Cell Science), and Kaitlyn Casimo (Allen Institute)

#

# **Learning Objectives**

1. **Define** the role of the endoplasmic reticulum
2. **Explain** the purpose and applications of high-throughput microscopy
3. **Compare** cell structures using the Allen 3D Cell Viewer
4. **Design** a future experiment to build on your findings

# **Anticipated Time**

1. Part I: ~ 15 minutes
2. Part II: ~ 20 minutes
3. Part III: ~ 20 minutes
4. Part IV: ~ 20 minutes

# **Part I. What has happened to these cells?**

Dr. G and his summer students want to discover a drug that could be used as an antibiotic against *Delftia acidovorans*, an opportunistic bacterial pathogen that has been found in drains, faucets, and water filtration systems. They used high-throughput (HT) drug screening to test a collection (“library”) of cancer drugs from the National Cancer Institute (NCI) and were excited to find a promising **hit**, a compound with potentially useful drug properties. This compound is highly potent at low doses against *Delftia acidovorans* and not cytotoxic when tested on mammalian cells. However, one of Dr. G’s students was learning from a friend in another lab how to use a stain to label the endoplasmic reticulum (ER) in live Chinese Hamster Ovary (CHO) cells, and she noticed that cells treated with low doses of their promising compound have **wildly** variable ER sizes and morphologies. She tells Dr. G., and together they search for information about ER structure. Dr. G. is a microbiologist and, along with his students, has limited cell biology experience.

Dr. G. and his students decide to use the Allen Cell Explorer open cell biology data as a reference dataset to compare to their drug-treated cells. This is a large, high-throughput dataset containing tens of thousands of cells. They hope to gain some useful insights about the human ER from this dataset.

## **Questions**

1. Draw a eukaryotic cell and identify the endoplasmic reticulum (ER). How is this structure different from a prokaryotic cell?

An example of a eukaryotic cell diagram can be found in Wiki Commons: <https://commons.wikimedia.org/wiki/Category:Endoplasmic_reticulum#/media/File:Endomembrane_system_diagram_en.svg>

Note the blebs and shape. Prokaryotic cells don’t have a nucleus and organelles, including the ER.

Representative student responses:


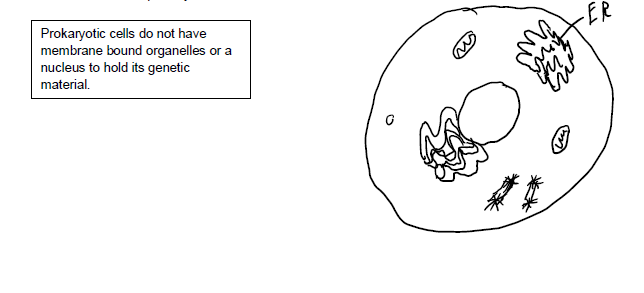


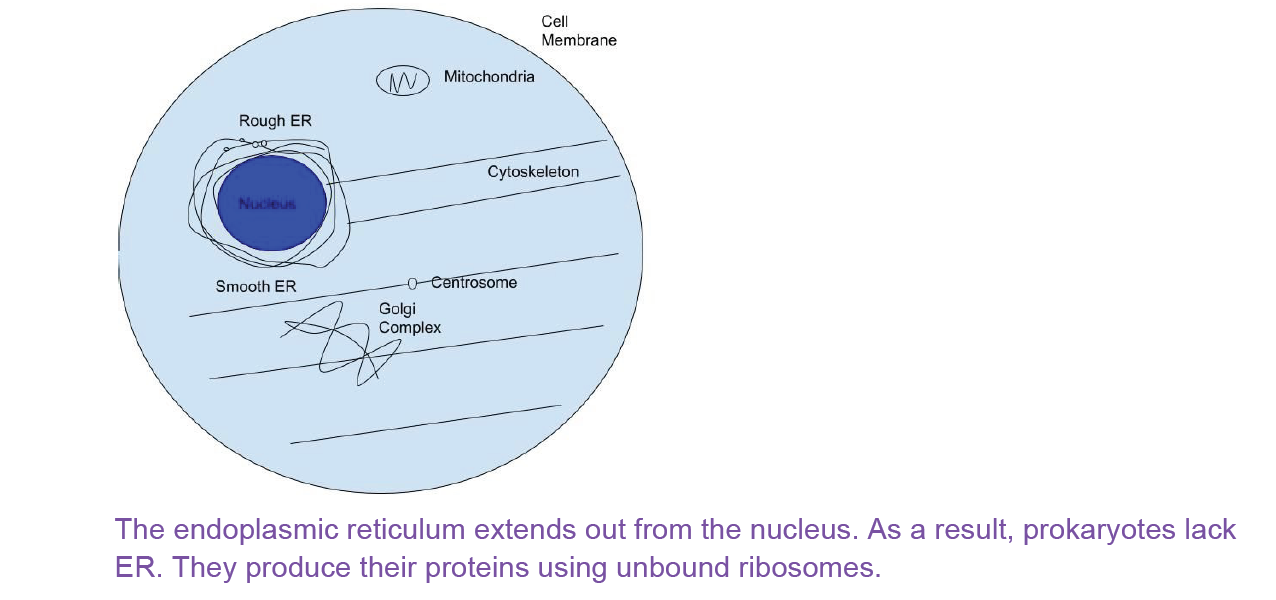


1. Demonstrate your knowledge: explain to the average adult the function of the ER in 1-3 sentences.

From Allen Cell Explorer: “The endoplasmic reticulum (ER) is a large membrane-bound network (or reticulum) consisting of tubules and flat compartments called cisternae. The network is continuous with the outer membrane of the nuclear envelope. The ER is the site of protein and lipid synthesis and calcium storage and release, among many other cellular functions.”

Helpful videos: [Endoplasmic Reticulum](https://www.youtube.com/watch?v=eH5k8XYKycs) (<https://www.youtube.com/watch?v=eH5k8XYKycs>, 2 min) and [Endoplasmic reticulum and golgi apparatus | Cells | MCAT | Khan Academy](https://www.youtube.com/watch?v=jDadorSbhi4) from Khan Academy (<https://www.youtube.com/watch?v=jDadorSbhi4>, 10 min)

A typical student response from the fall of 2020: “The ER is responsible for storing calcium in the cell, synthesizing proteins, and lipid metabolism.”

1. Demonstrate your knowledge: describe the shape of the ER you just drew.

Students will explain the shape of the ER they just drew in their own words.

#

# **Part II. This image does *not* look like the textbook image!**

Dr. G. and his students visit the Allen Cell Explorer [Visual Guide to Human Cells.](https://www.allencell.org/visual-guide-to-human-cells.html) This resource, along with other open data we will use in this case, has been produced by the [Allen Institute for Cell Science](http://cellscience.alleninstitute.org). They read about the Endoplasmic Reticulum (ER) and how researchers visualized the ER by **labeling Sec61-beta proteins** as discussed below.


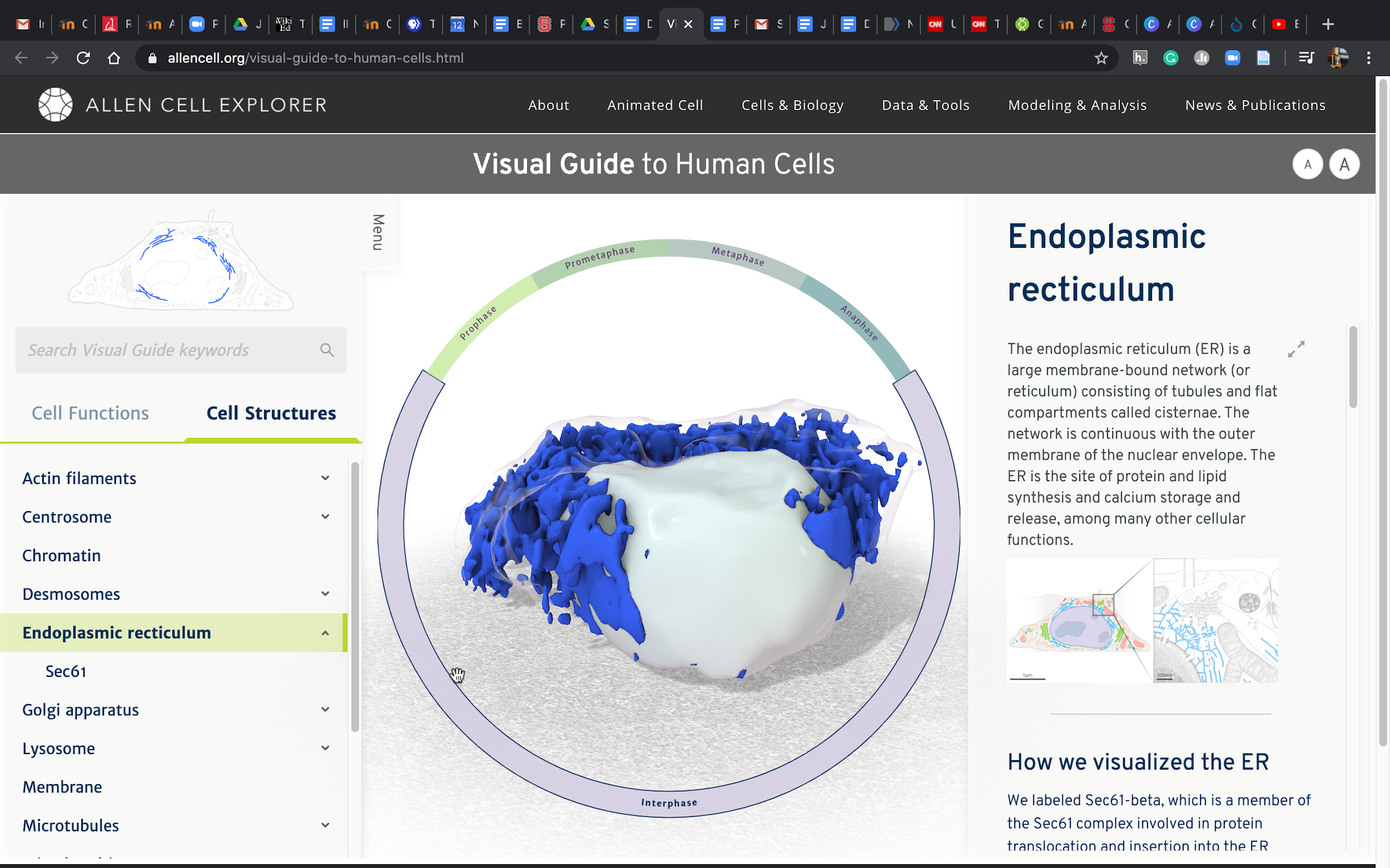


They then decide to view cells using the [**Allen Cell Feature Explorer**](https://cfe.allencell.org/) and visualize cells with the **Sec61-beta** protein tagged as shown next. Watch this 3-min [video](https://www.youtube.com/watch?v=UKXtEddLzjg) to learn more about the 3D Cell Viewer. These features are found in the Cell Feature Explorer, along with some additional tools for quantitative analysis of the cells. We’ll be concentrating on the cell images, but you may find additional insights using the graphing panel.

##

## **Your turn!**

Help Dr. G. and his students by visiting the Allen Cell Explorer resources. With the information you find, answer the questions below.

## **Questions**

1. Using the [Visual Guide to Human Cells](https://www.allencell.org/visual-guide-to-human-cells.html), look at the shape of the ER during the different phases of the cell cycle (prophase, prometaphase, metaphase, anaphase, and telophase/cytokinesis). Click on the **Menu** tab to reveal the cell structures and select ER. You can click on the **name** of the phase, and the visuals will change. You can drag the cell visual to reveal different perspectives. What changes do you notice? (in 2-3 sentences)

The ER position seems to change. Some phases resemble the textbook images, but others don't. Example student responses are presented below:

“During interface, the ER is packed on one side of the nucleus and it is packed together. During mitosis, the ER surrounds the nucleus and is less packed together, so it takes up more space”.

“In interphase and early mitosis, the ER is loose and distributed around the nucleus. As the nucleus prepares to divide at the end of mitosis, the ER condenses around it and ultimately is split between the two daughter nuclei.”

“This is interesting because it appears that the ER is mainly grouped to one side of the nucleus during interphase. But when the cell enters prophase and continues towards cell division, the ER begins to completely surround the nucleus. The ER continues to surround the nucleus as it divides during anaphase.”

“The variation in ER shape could be a result from wavy shape the ER takes during cell division. During cell division, the ER takes the shape of the nuclear envelope as it surrounds for the nucleus to split, resulting in the inconsistent shape.”

1. You notice under the **Morphology** section (panel on the right) the following text: “In hiPS cells, the ER is localized to the nuclear periphery and in tubules and sheet-like structures throughout the cytoplasm”. Dr. G’s students saw fluorescence using the ER-specific tag that **varied** in shape both within and between different cells. What explanation do you have for this? (2-3 sentences)

The cells may be in different phases or undergoing stress from treatment with the compound in the case of the CHO cells, in contrast to the unstressed, endogenously tagged hiPSCs. We expect students to think about potential reasons why the ER in their compound treated cells may be so variable. A student explanation could be:

“The intracellular variation is explained by the morphology section. It’s known that the ER can be found in multiple areas of the cell, but is localized to the nucleus. This implies it can take different shapes. The intercellular variation is probably the result of the cells being in different cell cycle phases. The ER’s shape changes depending on phase, typically condensing as the cell gets ready to divide.”

1. You then navigate to the [Allen Cell Feature Explorer](https://cfe.allencell.org), select **Sec61-beta** under Protein Tag from the menu on the left, and select several dots from the plot corresponding to cells. Images of cells will appear in your Gallery on the right, and you can click to visualize cells with fluorescently tagged Sec61-beta.

You click on a couple of cell images, keeping in mind that the green signal corresponds to a fluorescent tag for the ER, and the membrane and DNA are also stained. Do you notice any patterns? (2-3 sentences).

The Sec61-beta protein tag is used to visualize the ER. In different cells, the ER fluorescence surrounds the nucleus but the pattern does vary.

# **Part III. Different looking cells? Let’s get quantitative.**

You have been looking at cells for some time now and notice a wide **variety of patterns** for the Sec61-beta tagged cells. Furthermore, **it seems all cells look different!** This wasn’t what you are used to from your Cell Biology class in college... You want to quantify this as best you can.

In the Cell Feature Explorer, scroll back up to the plot to start measuring the cellular and nuclear volumes of the ER (tagged using the Sec61 protein tag).

## **Tasks & Questions**

1. Measuring cellular and nuclear volumes. Select **three cells** and compare the ER volumes. Record the approximate values.

We encourage instructors to try cellular and nuclear volume measurements on their own and do a quick demonstration or record a short video as an example. The Allen Cell Feature Explorer is intuitive and there are links to tutorials on this page: [Allen Cell Explorer](https://www.allencell.org/)

|  | **Cellular Volume** | **Nuclear Volume** |
| --- | --- | --- |
| **Cell 1** | **Answers will depend on cells observed** | **Answers will depend on cells observed** |
| **Cell 2** | **Answers will depend on cells observed** | **Answers will depend on cells observed** |
| **Cell 3** | **Answers will depend on cells observed** | **Answers will depend on cells observed** |

1. Compare your measurements with those obtained by other group members.

You want to learn more! You find: [The Integrated Mitotic Stem Cell](https://imsc.allencell.org/).

… and carefully scroll down and read… You use the 3D Cell Viewer embedded in the Integrated Mitotic Stem Cell page to visualize the ER and other structures superimposed in space and time.

##

## **Questions**

1. Do these images help you explain the variability in ER volumes you recorded previously? Think about the cell cycle and variability of other organelles.

Examples of student responses:

“Yes. They show how the size of the ER and nucleus can change dramatically throughout the cell cycle.”

“Yes, these images reveal how variable the cell volume and ER volume can be as the cells pass through different phases. Additionally, it can be seen that the ER disassembles and reassembles during the cell division process, which adds more variability.”

1. What do you find intriguing about the [The Integrated Mitotic Stem Cell](https://imsc.allencell.org/) web page and data?

Representative student responses:

“It is very interesting how the ER is translocated so that there is one on the side of the old nucleus and one on the side of the new nucleus. I would like to see a video of the new ER being made. Is it made in a new location or is the existing ER made bigger and then split in two?”

“How the microtubules seem to encompass the ER and nucleus. This makes sense given the impact microtubules have in cellular division.”

You start to wonder... **How are they visualizing the ER and other organelles?** You navigate to the [Methods for Microscopy page](https://www.allencell.org/methods-for-microscopy.html) to learn more about the process used by the Allen Institute and begin to read...

## **Questions**

1. Read the [Methods for Microscopy page](https://www.allencell.org/methods-for-microscopy.html). Summarize the methods used in no more than **five** sentences.

Responses will vary but must mention a large number of edited cell lines, automation used to seed plates and maintain lines, high-throughput microscopy to take thousands of images, image analysis with CellProfiles and Matlab. Ideally will also understand that cells are endogenously tagged with fluorescence, not stained, and that cell image files are stacks of 2D images that produce 3D data.


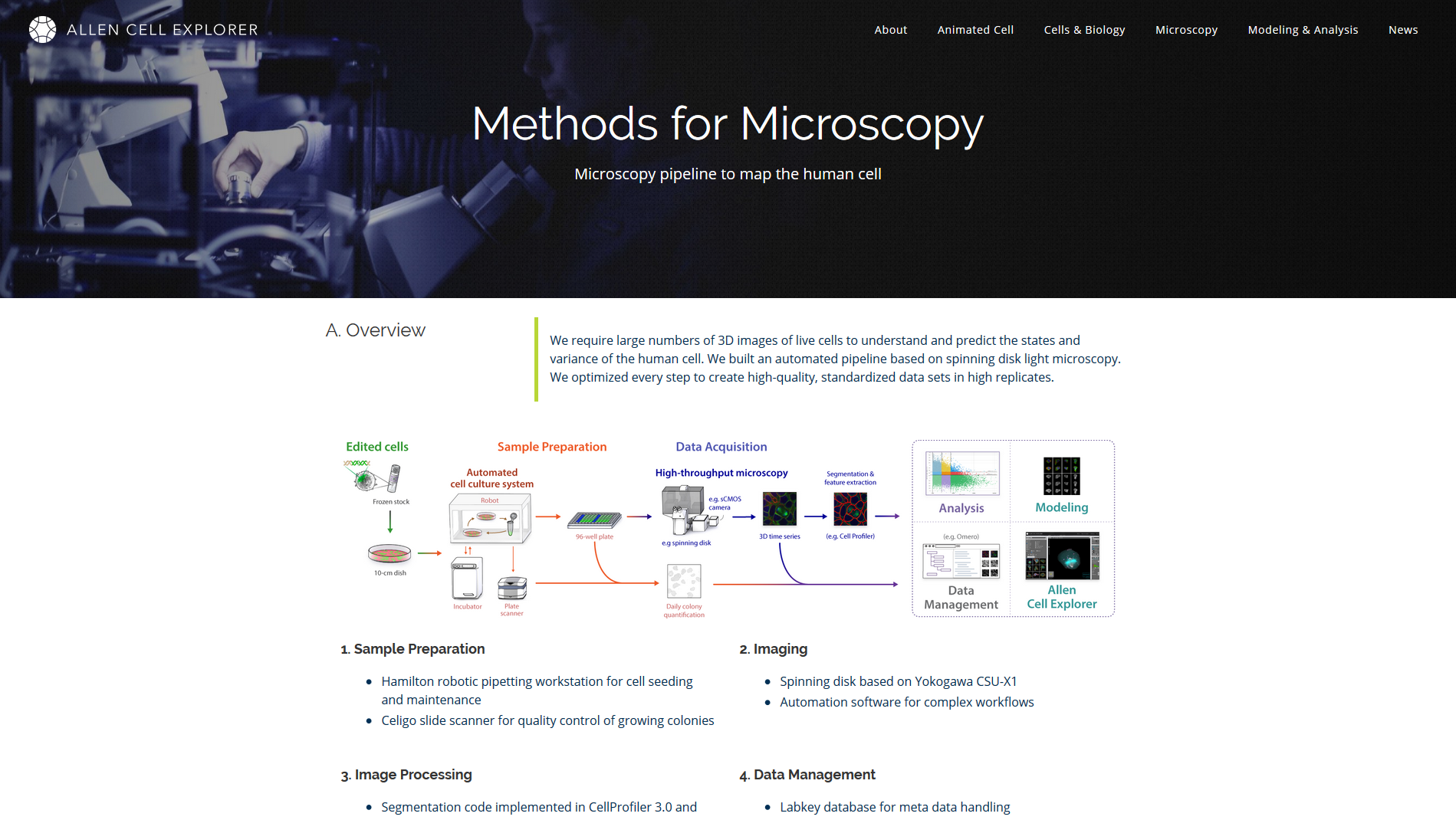


1. Think about Dr. G’s drug-treated CHO cells. What can we infer or visualize from the cells in the Allen Cell Explorer that we can’t do with the stained CHO cells?

- We can be confident that we are visualizing the ER in the Allen Cell Explorer cells, but we are uncertain how specific the stain used by Dr. G’s students is.
- Students may also contrast how we are visualizing the ER in each cell: a tagged ER protein is used in the Allen Cell Explorer hiPSC while a stain is used to visualize the ER in the CHO cells.
- Dr. G’s students are using CHO ovarian cells from a hamster, while the Allen Institute data uses (induced) stem cells from humans. A plus if students also mention that both experiments are *in vitro*.

1. What is one limitation of the endogenous fluorescence method used by the Allen Institute?

Possible answers include:

- Researchers can only tag one or two proteins at a time.
- Cells are imaged *in vitro*, not *in vivo*
- Though cells are heavily screened for effects on function, tag may in theory affect cell’s behavior

#

# **Part IV. Does this make sense, Dr. G?**

You have learned *a lot* from helping Dr. G’s students and visiting the Allen Cell Explorer. However, you still have to report back to Dr. G!

## **Questions**

1. Think about the **high-throughput microscopy** approach used by the Allen Institute and the tools developed to measure cell structures. What did you learn about the ER from the tools and site?

[Answers will vary. Rationale should be provided]

Typical student responses mention the variability of cell morphologies, the massive number of images captured using high-throughput microscopy approaches, and the opportunity to learn by mining open-source datasets such as these.

1. Think about the cells you viewed, what you learned about the ER, and the *Delftia* experiment. Do you think this high-content imaging approach will help Dr. G’s students? Why or why not? Think about the knowledge gained from this **approach** and the limitations of the dataset.

[Answers will vary. Rationale should be provided]

Some students say the difference in cell types and variability will not allow Dr. G’s students to obtain helpful information. Others think the data obtained from the high-content imaging experiments will help inform Dr. G about the natural variability of ER and then focus on more detailed experiments addressing the effect of their compound on ER morphology.

1. The reference data from the Allen Cell Explorer comes from human induced pluripotent stem cells, but Dr. G. and his student were using CHO cells in their drug screening study. What similarities are there between these two types of cells? What differences? Would you expect systematic variation between these two types of cells? Would you expect CHO cells to be more similar to hiPSCs than yeast cells are? Why?

The two cell types are different and may respond differently to the experimental compound.

1. If you could work with researchers at the Allen Institute, what would you do next? Design an **experiment** to build on your findings and help Dr. G. Describe the goal of the experiment, the methods and resources you will use, and the expected findings. Think critically about the potential limitations of your approach. Explain the experiment you design in 5-10 sentences. You may need to use external resources such as PubMed to find background research related to your experimental design.

[Answers will vary. Rationale should be provided]

The experiment may involve collecting new data not in the public datasets, or use only existing public data.

#

# **Reflection**

1. What was the most **memorable** concept or skill you learned from this case study?

[Answers will vary. Rationale should be provided] Representative student responses are provided below:

The use of CNN networks between imaging layers for the label-free predictive model was most fascinating to me as a data scientist. It allowed me to see a great biological imaging application for these networks!

The potential of a HT pipeline for image analysis and how it can expedite the research process.

1. What are you left **wondering** about [“**Nothing**” is an unacceptable answer]? How do you start the process of learning the answer?

[Answers will vary. Rationale should be provided] Representative student responses are provided below:

I wonder what the Golgi looks like in different stages of the cell cycle. To find this out high throughput microscopy should be used. The translocation protein for the Golgi should be labelled with a fluorescent protein. Then, imaging should be done.

I’m left wondering about what exactly determines the shape of the ER, and how this shape corresponds to the protein needs of the cell. I would begin to learn the answer by researching the mechanisms that follow initial replication based on cell-type and begin visualizations of these processes to see if it correlates with ER shape.
