# Supplementary material for: Does Organelle Shape Matter?: Exploring Patterns in Cell Shape and Structure with High-Throughput (HT) Imaging
Source: CourseSource. Author manuscript; Available in PMC 2022 Aug 17. (PMC9385133; doi:10.24918/cs.2022.3)
Supplement: S3 — Does Organelle Shape Matter? - Teaching Notes [file NIHMS1777492-supplement-S3.docx]

**
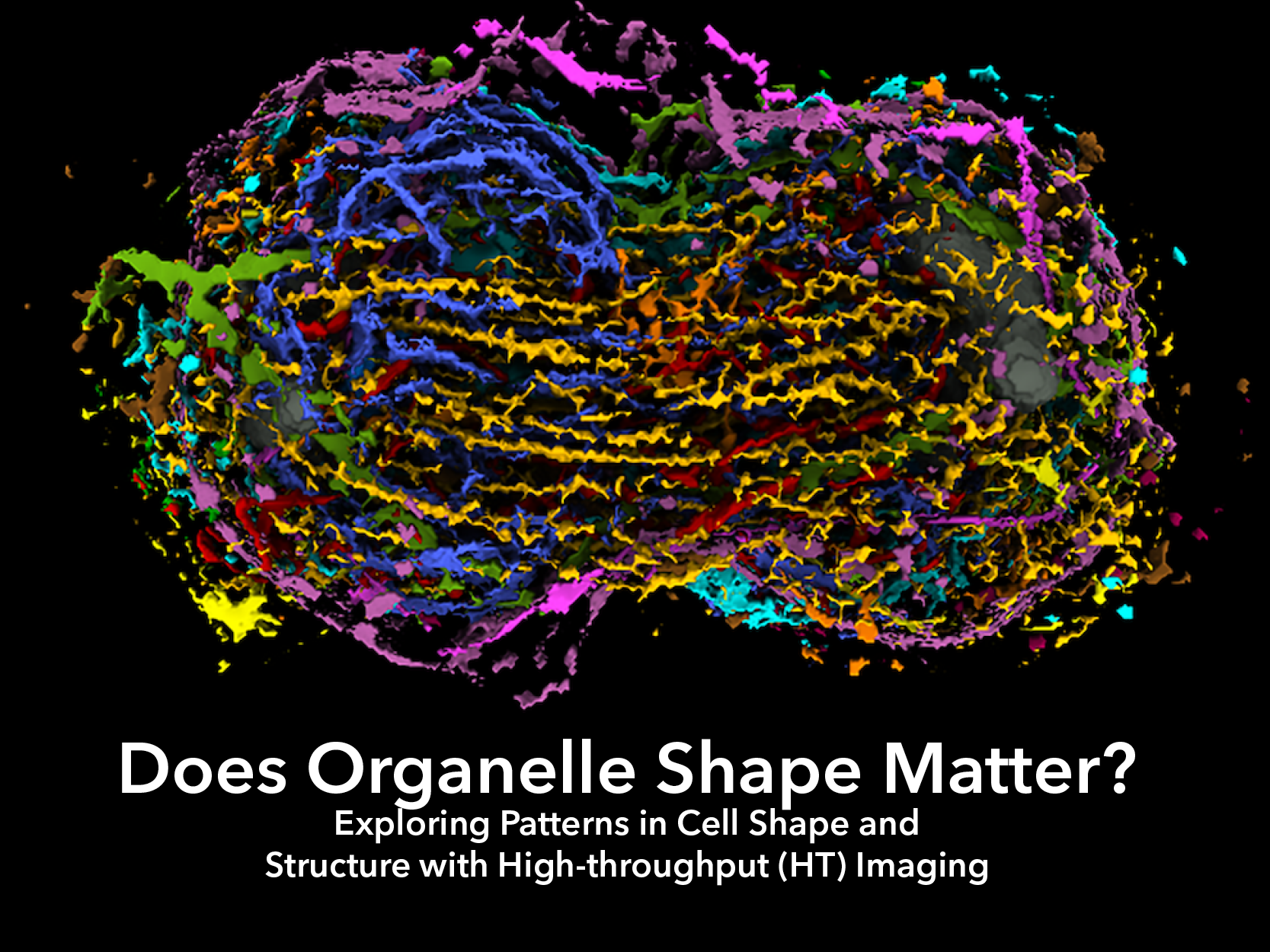
**

**Does Organelle Shape Matter:**

**Exploring Patterns in Cell Shape and Structure with High-throughput (HT) Imaging**

## **Teaching Notes**

Carlos C. Goller (North Carolina State University), Graham Johnson (Allen Institute for Cell Science), and Kaitlyn Casimo (Allen Institute)

#

# **Learning Objectives**

1. **Define** the role of the endoplasmic reticulum
2. **Explain** the purpose and applications of high-throughput microscopy
3. **Compare** cell structures using the Allen 3D Cell Viewer
4. **Design** a future experiment to build on your findings

# **Suggested Script**

It is important to **contextualize** the case when introducing it to students to raise awareness of the purpose and significance of these activities. This can be done by highlighting how the case introduces **high-throughput microscopy methods and open datasets** using a realistic scenario. Discussion of the Allen 3D Cell Viewer website and available resources before assigning the case allows students to prepare for the activity. Setting expectations for the depth of responses and work required will help students estimate the time they need to devote to successfully complete the assignment. If working in groups is allowed, clear guidelines about the number of students per group, the composition of teams, and how they will submit their completed case worksheet need to be addressed prior to and during the case.

An example script to introduce this case in an upper-division undergraduate and graduate molecular biology course is presented below. This script can be modified to align with the course outcomes and activity objectives for your course, as well as the level of assessment of the student responses.

## Introduction

Class:

We have learned about high-throughput screening of small molecules to develop new human therapeutics in past sessions. These techniques allow researchers to analyze hundreds of thousands of different molecules and conditions in assays, but the experimental design must be robust enough to be able to distinguish between signal and background noise of positive and negative controls, for example. What is background noise/signal?

High-throughput approaches are not limited to drug screening and the use of automation in molecular biology. Automated microscopy has helped researchers create hundreds of thousands of images to learn about basic biological processes, cell structure, and drug discovery.

We have designed a case study that introduces the concept of **high-throughput or high-content microscopy using the tools created by the Allen Institute** and available on the Allen Cell Explorer website: <https://www.allencell.org/>

The Allen Institute has a tremendous wealth of resources on this site including thousands of images of engineered cells that allow us to learn about cell variability and subcellular structures.

Be prepared: we will challenge you to review your cell biology and notions of organelle shape. We will analyze large microscopy datasets to learn about the variability of organelles and, importantly, the use of innovative high-throughput techniques to uncover fundamental cell biology knowledge.

For this case study, you will work in small groups with three or four members. All members are expected to work together as a team and complete the case study worksheet. Worksheets will be turned in at the conclusion of the case study and graded based on completeness. We will also call on groups to share their findings. Therefore, you will work together to reach a common consensus and elect a spokesperson for the group.

Be prepared to use your web browser to analyze images of cells on the Allen Cell Explorer site and search for published studies that support or reject your hypothesis. Importantly, remember to think critically about the methodology used by both the researchers at the Allen Institute and Dr. G (and his students). High-throughput Discovery encompasses a wide and growing range of techniques that enable scientists to harness the power of multiplexed technology!

We look forward to productive discussions about this case study!

## Class-level Discussions

After each part of the case study, questions can be presented during in-person sessions. Students can use hard copies of the case study to take notes and discuss in small groups before class-level discussions. The goal of these brief (3-10 minute) conversations is to share ideas and correct misconceptions. Pausing also allows participants to catch up or share questions with other groups and instructors. Alternatively, participants can respond using student response systems, including Top Hat or Poll Everywhere.

Think-pair-share can be used for students to elaborate on their responses and share their reasoning with their peers. The conversations after each part of the case are truly valuable to reinforce concepts and help students learn how to navigate the website.

In online asynchronous environments, case study questions can be assigned to be due before opening discussion forums with prompts for students to reflect on their case study experience and findings.

## Challenges, Wrap-up, and Reflection

A common difficulty students encounter is understanding the difference between stains and endogenously tagged proteins as used for microscopy in this case study. We often have to correct student wording and emphasize what they are looking at when we say that they are visualizing the ER, for example.

One possibility for wrap-up discussions is for instructors to provide students with a video and audio summary of the lesson and review the learning objectives. Loom.com, Screencast-o-matic (<https://screencast-o-matic.com/>), or video/content capture features often available within your learning management system (LMS) can be used to create a video. Videos should include captions, and the Google Read and Write (<https://www.texthelp.com/>) extension can be used to create an audio .mp3 of the documents (case, key, and class feedback). Providing accessible documents and information for the lesson in multiple modalities (audio, video, and text) encourages participation by all.

# **Useful Resources**

## Allen Institute Visual Guide to Human Cells:

<https://www.allencell.org/visual-guide-to-human-cells.html>

A resource developed specifically for students that pairs a 3D cellular model with detailed explanations of cell structures and functions. The explanations are geared towards an advanced high school or early college biology student. A tutorial is also available at <https://www.allencell.org/visual-guide-tutorial.html>.

## Methods for Cells in the Lab:

<https://www.allencell.org/methods-for-cells-in-the-lab.html>

This web page includes illustrations and explanations about the development of the cell lines as well as standard operating procedures for using the cells in a lab. These are the same cell lines shown in the images available in the 3D Cell Viewer and Cell Feature Explorer.

## Instructional Videos and Tutorials for Cell Methods:

<https://www.allencell.org/instructional-videos-and-tutorials-for-cell-methods.html>

These videos illustrate the standard operating procedures seen in the documents at Methods for Cells in the Lab.

## Methods for Microscopy:

<https://www.allencell.org/methods-for-microscopy.html>

This page includes methods that highlight the use of automated microscopy and cell culture to generate large image datasets.

## Allen Cell Discussion Forum:

<https://forum.allencell.org/>

The Allen Institute for Cell Science open discussion forum. Students and instructors can browse past questions and answers, or post their own questions.
